# Муковисцидоз как полиэндокринное заболевание (обзор литературы)

**DOI:** 10.14341/probl12694

**Published:** 2021-03-30

**Authors:** Н. Б. Чагай, Г. Я. Хайт, Т. М. Вдовина, А. А. Шафорост

**Affiliations:** Ставропольский краевой клинический консультативно-диагностический центр; Ставропольский государственный медицинский университет; Ставропольский краевой клинический консультативно-диагностический центр; Ставропольский государственный медицинский университет; Ставропольский краевой клинический консультативно-диагностический центр; Ставропольский краевой клинический консультативно-диагностический центр

**Keywords:** CFTR, муковисцидоз, муковисцидоз-ассоциированный сахарный диабет, остеопороз, врожденная двусторонняя аплазия семявыносящих протоков, необструктивная азооспермия

## Abstract

Ген CFTR (cystic fibrosis transmembrane regulator) кодирует синтез одноименного белка, который функционирует как прямой активатор анионного транспорта. Cl– является наиболее распространенным анионом, в качестве антагониста катионов натрия и калия обеспечивает электронейтральность клеточных мембран в покое, вместе с катионами служит важным осмолитом и формирует поток воды через клеточные мембраны для трансэпителиальной секреции. Железистые клетки при муковисцидозе (МВ) задерживают Cl– и Na+, а выделяемый ими секрет отличается чрезмерной вязкостью. Субнормальная активность CFTR приводит к застою мукоцилиарного клиренса, торможению кишечного транспорта.Помимо экзокринных нарушений, мутации CFTR ассоциируются с уменьшением объема, массы, усилением апоптоза β-клеток поджелудочной железы, значительным подавлением экзоцитоза инсулина в ответ на стимуляцию глюкозой и глюкагоноподобным пептидом-1, гиперглюкагонемией на фоне дефекта подавления инсулином функции α-клеток, но снижением максимальной емкости α-клеток.Дефицит и прогрессирующее снижение минеральной плотности костной ткани являются ожидаемыми вторичными проявлениями МВ ввиду панкреатической экзокринной недостаточности с мальабсорбцией питательных веществ и жирорастворимых витаминов. Но у пациентов с мутацией F508del установлены значимое уменьшение синтеза в остеобластической формации OPG, COX-2, PGE2, повышение активности антианаболического NF-kB. Речь идет о дефекте канонического сигнального пути (Wnt/β-катенин), который регулирует экспрессию генов-активаторов остеобластогенеза, разобщении этапности физиологического костного ремоделирования.Помимо врожденной двусторонней или односторонней аплазии семявыносящих протоков, увеличение частоты мутаций CFTR также обнаруживается при необструктивной азооспермии, олиго-, астено- и тератоспермии. CFTR участвует во входе HCO3– в клетки Сертоли для запуска cAMP-зависимой транскрипции, и его дефекты приводят к подавлению ФСГ-зависимой экспрессии генов сперматогенеза, потере последовательности в каскаде Wnt, разрушению зависимого от PGE2 трансэпителиального взаимодействия и, как следствие, гематотестикулярного барьера.МВ характеризуется, наряду с классическими признаками, эндокринной дисфункцией поджелудочной железы, остеопорозом с подавлением остеобластогенеза, дефектом сперматогенеза.

## Abstract (en)

The cystic fibrosis transmembrane regulator (CFTR) gene encodes the synthesis of a protein of the same name, which functions as a direct activator of anionic transport. Chloride is the most abundant anion; as an antagonist of Na+ and K+, it provides electroneutrality of cell membranes at rest; together with cations, it serves as an important osmolyte and forms water flow across cell membranes for transepithelial secretion.Glandular cells in CF trap Cl– and Na+, and the prodused secretion is excessively viscous. Subnormal CFTR activity leads to stagnation of mucociliary clearance, inhibition of intestinal transport.In addition to exocrine disorders, CFTR mutations are associated with a decrease in volume, mass, increased apoptosis of β-cells of the pancreas, a significant suppression of insulin exocytosis in response to stimulation with glucose and glucagon-like peptide-1, hyperglucagonemia against the background of a defect in the suppression of α-cell function by insulin, but a decrease in maximum capacity α-cells.Deficiency and progressive decline in bone mineral density is an expected secondary manifestation of CF due to pancreatic exocrine insufficiency with malabsorption of nutrients and fat-soluble vitamins. However, in patients with the F508del mutation, a significant decrease in the synthesis of OPG, COX-2, PGE2 in the osteoblastic formation, and an increase in the activity of the antianabolic NF-kB were found. We are talking about a defect in the canonical signaling pathway (Wnt/β-catenin), which regulates the expression of genes-activators of osteoblastogenesis, dissociation of the stages of physiological bone remodeling.In addition to congenital bilateral or unilateral aplasia of the vas deferens, an increase in the frequency of CFTR mutations is also found in non-obstructive azoospermia, oligo-, astheno- and teratospermia. CFTR is involved in the entry of HCO3– into Sertoli cells to trigger cAMP-dependent transcription and its defects lead to suppression of FSH-dependent gene expression of spermatogenesis, loss of sequence in the Wnt cascade, destruction of the PGE2-dependent transepithelial interaction and, as a consequence, the blood-testicular barrier.CF is characterized, along with classical signs, by endocrine dysfunction of the pancreas, osteoporosis with suppression of osteoblastogenesis, and a defect in spermatogenesis.

Муковисцидоз (МВ) является распространенным моногенно-наследуемым заболеванием [1], история изучения которого начинается с момента описания в 1905 г. австрийским иммунологом К. Landsteiner деструктивных изменений в поджелудочной железе (ПЖ) у двух детей с мекониальной непроходимостью [2]. Именно по этой причине первоначально болезнь назвали «кистозным фиброзом». Fanconi G. et al. в 1936 г. обратили внимание на связь патологии ПЖ с наличием аномально вязкого секрета в бронхах. «Пробки» из мокроты у пациентов с хроническим кашлем существенно облегчали выявление болезни, став ее ключевым симптомом, поэтому многие специалисты уже в начале 1940-х гг. предпочли данному состоянию название mucoviscidosis. В 1949 г. американский педиатр D. Andersen заметил, что пациенты с МВ подвержены развитию теплового шока (прострации) вследствие дегидратации. Настоящим открытием стало обнаружение патологии потовых желез как источника потери соли и обезвоживания [3]. В это же время был предложен тест на определение концентрации Na+ и Cl– в потовой жидкости, но для получения больших ее объемов применяли способ обматывания тела пластиковой пленкой, создавая угрозу гиперпирексии. Модифицированная Гибсоном–Куком в 1959 г. потовая проба, основанная на определении концентрации хлоридов биохимическим методом, до сих пор остается золотым стандартом прижизненной диагностики МВ [1]. В 1968 г. Kaplan E. et al. заметили [4], что взрослые мужчины с МВ бесплодны из-за отсутствия семявыносящего протока, а гистологическое исследование ткани яичек выявило изменения в сперматогенезе, часть сперматозоидов с признаками аномалии.

Так, к 70-м гг. ХХ в. МВ представлялся мультисистемным заболеванием с поражением дыхательных путей, желудочно-кишечного тракта и репродуктивной системы у мужчин. Однако только в результате открытия в 1989 г. гена МВ, регулятора трансмембранной проводимости (cystic fibrosis transmembrane regulator, CFTR) [5], стали понятны этиопатогенетические механизмы и причины клинического разнообразия данного заболевания.

Ген CFTR локализован в середине длинного плеча 7 аутосомы (7q31.2), содержит 27 экзонов и охватывает 250 000 пар нуклеотидов. Ген кодирует синтез одноименного белка на рибосомах эндоплазматического ретикулума большинства эпителиальных клеток (в потовых, слюнных, железах в бронхах, ПЖ, кишечнике, урогенитальном тракте) [1]. CFTR перемещается по секреторному пути к клеточной поверхности, где располагается в билипидном слое мембраны и функционирует как прямой активатор анионного транспорта [6].

Выделено около 2000 мутаций, ответственных за развитие симптомов МВ. Наиболее часто встречающиеся в России: F508del (52,79%), СFTRdele2,3 (6,32%), E92K (2,65%), 2184insA (2,02%), 3849+10kbC>T (1,65%), 2143delT (1,65%), G542X (1,33%), N1303K (1,33%), W1282X (1,11%), L138ins (1,06%) [1]. В кавказских популяциях МВ встречается с частотой 1 на 2500 живорождений. По разным оценкам, во всем мире МВ больны 70–100 тыс. человек [7] (http://www.genet.sickkids.on.ca/cftr/). Генетический полиморфизм, более высокий риск ассоциируемых с МВ нарушений у гетерозиготных носителей мутации CFTR, низкая частота встречаемости большинства мутаций и их компаундное состояние [8] объясняют чрезвычайную распространенность и выраженное фенотипическое разнообразие заболевания, от тяжелых до субклинических форм. Существенное влияние на степень тяжести и клиническую вариабельность заболевания у лиц с одинаковыми мутациями CFTR оказывают гены-модификаторы — гены, отличные от CFTR, в большинстве кодирующие ионные каналы и/или обмен ионов [9].

CFTR представляет собой крупный белок (1480 аминокислот), разделенный на 2 гомологичные половины, каждая из которых содержит 6 охватывающих мембрану сегментов и нуклеотидсвязывающие домены (NBD1 и -2), связанные регуляторным (R) доменом (рис. 1). Большая часть белка погружена в цитоплазму (77%), 19% его находится в мембране, 4% — внеклеточно [10].

**Figure:**
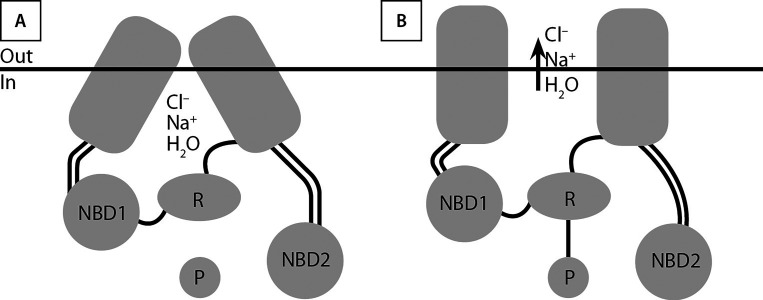
Рисунок 1.Регулятор трансмембранной проводимости (CFTR): закрыт (А), открыт (В).

Открытие канала — двухэтапный процесс, включающий фосфорилирование и связывание/гидролиз аденозинтрифосфата (adenosine 5’ trisphosphate — ATP). Фосфорилированию (активации) — присоединению фосфатной группы к гидроксильной группе боковой цепи остатка серина — подвергается R-домен, и это служит запуском цикла открытия-закрытия канала CFTR. Связывание и гидролиз первой молекулы ATP в NBD1 вызывает открытие канала, поток ионов хлора устремляется по электрохимическому градиенту и зависит от разницы их концентраций во внутри- и внеклеточном пространстве. Гидролиз второй молекулы ATP в NBD2 обеспечивает закрытие CFTR [10]. CFTR отвечает за ход во внеклеточное пространство ионов Cl– и увлекаемое за ними линейное движение воды, но также регулирует активность мембранных белков, включая эпителиальный натриевый канал (epithelial sodium channel, ENaC), обменник Cl–/HCO3– и др. [7].

Хлорид является наиболее распространенным анионом и выполняет множество различных биологических функций. Будучи антагонистом Na+ и K+, ион Cl– обеспечивает электронейтральность клеточных мембран в покое, вместе с положительно заряженными катионами служит осмолитом, формирует поток воды через клеточные мембраны для баланса объема клеток, трансэпителиальной секреции или абсорбции жидкости, может играть «химическую» роль, связываясь с белками, изменяя их активность [11].

Открытие канала CFTR в норме сопряжено с выходом физиологического по содержанию воды секрета в просвет бронхов, панкреатические протоки, кишечную трубку. При МВ железистые клетки задерживают Cl– и Na+, а выделяемый ими секрет отличается чрезмерной вязкостью. Субнормальная активность CFTR приводит к застою мукоцилиарного клиренса, жизненно важного для поддержания здоровья легких, торможению кишечного транспорта.

CFTR обеспечивает синтез антиоксиданта глутатиона, необходимого для детоксикации активных форм кислорода. Дефект CFTR сопровождается развитием оксидативного стресса локально в легких, нарушением фагоцитарной функции в альвеолах и снижением противодействия патогенным микроорганизмам [12]. Мукостаз в респираторном тракте осложняется обструктивным бронхитом с хронической бактериальной колонизацией, изменением архитектоники легкого с появлением бронхоэктазов, развитием фиброза [6].

В ПЖ ввиду замедления тока пищеварительного секрета, осаждения белковых преципитатов формируется обструкция протоков. Синтез активных панкреатических ферментов в этих условиях завершается аутолизом ткани, вследствие чего тело органа трансформируется в скопление кист с последующим фиброзированием [6].

В потовых железах хлорный канал функционирует несколько иначе и обеспечивает реабсорбцию Cl– при движении пота вдоль протоков. Поэтому повышение концентрации хлорида в потовой жидкости без явных патологических изменений в самих железах [6] — патогномоничный признак МВ [3].

Любые ионные каналы представляются трансмембранными белками, имеющими «пору», которая обеспечивает диффузию определенных ионов через градиент концентрации. Это могут быть антипортеры (обменники), симпортеры (котранспорт в одном и том же направлении) и насосы (использование энергии от гидролиза ATP) [13].

К настоящему времени выделены три основных структурных класса хлоридных каналов. Первый — лиганд-активируемые Cl–-каналы, в качестве мессенджера использующие ионы кальция. CFTR является единственным членом хлоридного канала второго класса, относится к семейству ATP-связывающих кассетных (ABC) транспортеров, то есть управляемых энергией ATP. Последний, третий, класс образован семейством ClC (Cl–-каналы), которые имеют около 12 трансмембранных доменов, каждый мономер снабжен порами (каналы с двойными стволами) и функционирует как стробированный канал. Семейство ClC включает по меньшей мере девять различных членов. Предполагается, что каналы ClC-0, -1, -2, Ka, Kb являются преимущественно каналами плазматической мембраны, тогда как ClC-3, -4, -5 и ClC-6, -7 в основном выполняют свои функции во внутриклеточных мембранах [14].

Предполагается, что определенные ClC могут служить альтернативными путями секреции эпителиального хлорида при МВ. Секреторные гранулы, по-видимому, снабжены специфическими наборами ионных каналов, которые представляют собой идеальные мишени для разработки препаратов с высокой тканевой или клеточной специфичностью и селективным способом действия. Но до сегодняшнего дня не выявлено эффективных высокоаффинных блокаторов или активаторов хлорных каналов, способных заменить CFTR [11].

Клиническая гетерогенность МВ в значительной степени зависит от «тяжести» мутаций CFTR [15]. Мутации класса I определяют полную блокаду синтеза CFTR, обнаруживаются в 2–5% случаев МВ во всем мире. Дефекты класса II сопровождаются аномалией процессинга белка, что приводит к его аберрантной локализации, нарушению транспорта к апикальной мембране. Приблизительно 50% пациентов МВ гомозиготны, а 90% гетерозиготны по аллелю F508del (наиболее распространенный в этом классе и в целом в популяции). Мутации класса III приводят к поломке в R-домене, резко снижают скорость ATP-зависимого открытия CFTR [16]. Мутации I–III класса ассоциированы с классическим МВ, наличие двух любых из них в гомозиготном или компаундном состоянии всегда ассоциируется с панкреатической недостаточностью, тяжелым поражением легких [8].

Мутации класса IV вызывают нарушения проводимости CFTR и измененную частоту потока ионов; класса V — нарушение стабильности мРНК или зрелого белка CFTR; класса VI — ускорение его распада из-за аномалий С-терминального фрагмента. Мутации IV–VI классов называют «мягкими» ввиду сохранения остаточной функции ПЖ [8][16].

Принято различать классический МВ с панкреатической недостаточностью (легочно-кишечная форма), с ненарушенной функцией ПЖ (преимущественно легочная форма), а также заболевания, ассоциированные с геном CFTR, к которым относятся изолированная обструктивная азооспермия, хронический панкреатит, диссеминированные бронхоэктазы [1].

Сегодня благодаря разработке эффективных методов диагностики МВ стали появляться сведения о патофизиологии нарушений функции органов и тканей, ранее не в полной мере объясняемые дефектом трансмембранного регулятора проводимости ионов. Необходимость квалифицировать «нетипичных» пациентов с доказанной CFTR-дисфункцией привела к определению концепции неклассического МВ и появлению термина «муковисцидоз-ассоциированные состояния» [1]. При атипичном МВ респираторные симптомы могут не проявляться до зрелого возраста, но все же характерны рецидивирующая пневмония, прогрессирующая обструкция бронхов, идентифицированная как астма или хроническая обструктивная болезнь легких. Пациенты могут страдать от хронического рино- или пансинусита, поражения слюнных желез, синдрома холестаза [17].

Сахарный диабет и остеопороз являются внелегочными осложнениями МВ, однако патофизиология этих состояний многофакторна, в полной мере не изучена и сегодня предполагает прямую зависимость от CFTR прогрессирующего нарушения функций островкового аппарата ПЖ и костного метаболизма. Мужское бесплодие, как наряду с клиническими проявлениями МВ, так и изолированное, ранее изученное как вариант обструктивной азооспермии, также интересно с точки зрения сведений о патологии собственно сперматогенного эпителия. В представленном обзоре будут рассмотрены основные механизмы развития, принципы диагностики и терапии сахарного диабета (СД), ассоциированного с МВ (МВСД), остеопороза и мужского бесплодия при мутациях гена CFTR.

## МУКОВИСЦИДОЗ И САХАРНЫЙ ДИАБЕТ

Первое упоминание о нарушениях метаболизма глюкозы среди больных МВ относится к 40–50-м гг. XX в. [18].

Ранее МВСД считался крайне редким явлением, что было обусловлено малой продолжительностью жизни больных. В настоящее время он диагностируется у 2% детей, 19% подростков и 50% пациентов старше 30 лет [19], достоверно чаще встречаясь при «тяжелых» мутациях [20]. Для МВ характерны аномально высокие показатели гликемии в промежуточных отрезках времени при проведении глюкозотолерантного теста (перемежающаяся, бессимптомная гипергликемия), не подчиняющиеся стандартным общепринятым критериям СД [21].

Деструкция ПЖ вследствие экзокринных нарушений, прогрессирующего фиброза и, соответственно, паракринное воспаление с повышением свободнорадикального синтеза являются причинами уменьшения массы островкового аппарата и нарушения секреции инсулина [7]. По данным Bogdani et al. [22], в островках имеет место иммунная инфильтрация, представленная лейкоцитами (Т-клетки CD8+ и CD4+), но не макрофагами. Патология островков при МВ не сравнивается с глубокой аутоиммуноопосредованной деструкцией β-клеток, наблюдаемой при СД 1 типа, но локализованное воспаление указывает на вероятность экспрессии провоспалительных цитокинов и ответственно за угнетение выживаемости β-клеток [21]. Мутации CFTR соотносятся с уменьшением объема, массы [23], усилением апоптоза β-клеток [24]. Ацинарная деструкция, сопровождаемая инфильтрацией воспалительными клетками, замещением жирами, появлением обширного фиброза и кистозной дилатации крупных протоков, какое-то время сосуществует с сохраненной эндокринной частью органа. Но постепенно прогрессирует деструктуризация островков на фоне неблагоприятного микроокружения [21][25]. Предполагается, что значительная потеря (более 50%) массы β-клеток сопряжена с секреторной дисфункцией и недостаточностью, именно они определяют величину наблюдаемого дефицита секреции инсулина. Вероятно, терапия МВСД должна быть направлена на улучшение высвобождения инсулина функционирующими β-клетками [21].

Koivula F.N.M. et al. [19] обнаружили при МВ изменения в морфологии β-клеток до развития собственно диабета или экзокринного фиброза. CFTR экспрессируется в β-клетках человека, одновременно регулируя активность другого хлоридного канала Anoctamin 1. Ингибирование CFTR вызывает значительное снижение экзоцитоза инсулина в ответ на стимуляцию агонистами циклического аденозинмонофосфата (cAMP) (глюкозозависимая секреция) и глюкагоноподобным пептидом-1. Четко определяется дефект деполяризации клеточной мембраны, соответствуя нарушению первой фазы секреции инсулина [26][27]. При мутации F508del определяются уменьшение площади β-клеток с сохранением их количества, повышенная секреция проинсулина, но при снижении продукции С-пептида. Это, вероятно, стоит трактовать как дефект расщепления проинсулина и выход незрелого инсулина. Экзоцитоз и количество пристыкованных к мембране гранул уменьшено [28] (рис. 2).

**Figure:**
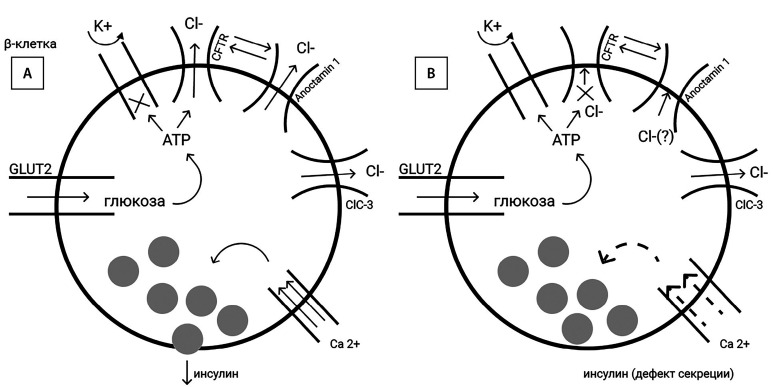
Рисунок 2. Регуляция секреции инсулина при функционирующем (А) и дефектном CFTR (В).

Закрытие ATP-чувствительных K+-каналов (KATP) в ответ на повышение гликемии считается начальным событием, которое деполяризует мембрану β-клеток и активирует потенциалзависимые Ca2+-каналы, далее увеличивается концентрации внутриклеточного Ca2+ с последующим экзоцитозом инсулина. Best L. et al. показали [29], что индуцированная глюкозой электрическая активность в β-клетках также зависит от внутриклеточной концентрации Cl–, то есть используется дополнительный анионный механизм запуска экзоцитоза инсулина. При этом ответственный канал Cl– остается нераспознанным. CFTR — это cAMP/протеинкиназа-А(cAMP/PKA)-зависимый Cl–-канал, контролируемый внутриклеточным ATP, получаемым при метаболизме глюкозы. Возможно, открытие CFTR активируется именно глюкозой. Подтверждением этой гипотезы является регистрируемый на мембране и зависимый от оттока Cl– потенциал действия, как по амплитуде, так и по частоте соотносимый с осцилляциями Ca2+, мембранной деполяризацией и, следовательно, секрецией инсулина. Не решено, является ли CFTR непосредственно управляемым глюкозоиндуцированными изменениями ATP либо глюкозозависимыми киназами, которые могут фосфорилировать CFTR [30].

Известно, что в β-клетке существует ряд других хлорных каналов [23]. В частности, ClC-3-канал экспрессируется на мембране секреторной гранулы, участвует в секреции инсулина, но не рассматривается как ведущий в глюкозозависимой деполяризации мембраны [30].

Показано, что CFTR негативно регулирует продукцию глюкагона путем потенцирования ATP-чувствительных калиевых каналов. A-клетки снабжены ко-транспортером KCl, поддерживающим низкий уровень хлорида в клетке. Открытие CFTR вызывает проникновение ионов K+ и Cl– с последующей гиперполяризацией мембраны и ингибированием секреции глюкагона [31]. Имеются сведения об увеличении численности α-клеток у пациентов с МВ как с диабетом, так и без него [21]. Мутация F508del, по данным Edlund A. et al. [28], ассоциируется с гиперглюкагонемией, то есть CFTR участвует в регуляции потенциала покоя/действия α-клеток. Неопределенность в том, что дефект подавления глюкагона во время нагрузки глюкозой сочетается с нарушением регуляции его высвобождения. Cекреция глюкагона, индуцированная гипогликемией, снижена как при МВ с нормальной толерантностью к глюкозе, так и у пациентов с МВСД [28]. При наличии мутации F508del количество глюкагона, секретируемого из островков в течение 15 минут и 1 ч, сопоставимо, что указывает на низкую максимальную емкость α-клеток [21].

Соматостатин секретируется δ-клетками, в норме ингибируя продукцию инсулина и глюкагона посредством паракринных эффектов. У пациентов с МВСД по сравнению со здоровыми субъектами уровень соматостатина в плазме повышается после стимуляции аргинином, хотя неизвестно происхождение избытка этого гормона (секреция из островков или других соматостатинсекретирующих клеток кишечника) [21]. CFTR не присутствует в δ-клетках человека, поэтому, возможно, повышенная секреция глюкагона повышает продукцию соматостатина. Усиление присутствия соматостатина является дополнительным фактором дефицитной секреции инсулина при МВ [28].

Таким образом, структурные, ауто- и паракринные нарушения всех клеточных групп островкового аппарата ответственны за развитие МВСД.

В качестве скрининга для своевременной диагностики МВСД рекомендовано проведение стандартного перорального глюкозотолерантного теста [1]. Но самые ранние нарушения метаболизма углеводов выявляются при непрерывном мониторинге глюкозы, более половины больных МВ с невыявленным нарушением толерантности к глюкозе демонстрируют колебания гликемии в основном после приема пищи >11,1 ммоль/л. Толерантность к глюкозе со временем прогрессирует до МВСД без гипергликемии натощак, а далее — с гипергликемией натощак [20]. Коморбидность МВ и диабета утяжеляет представительство патогенной бактериальной флоры в дыхательных путях и, соответственно, прогнозы по выживаемости пациентов. Смертность у пациентов с МВСД в 6 раз выше, чем при МВ, не отягощенном нарушением углеводного метаболизма [7].

На важную роль генетических модификаторов в увеличении риска развития МВСД указали данные о его частоте в семьях с отягощенным анамнезом по СД 2 типа [32]. Исследование общегеномных ассоциаций (GWAS), международный консорциум модификаторов генов МВ выявили генетические варианты 5’некодирующих регионов гена SLC26A9, которые ассоциированы с возрастом начала МВСД. SLC26A9 функционирует как обменник Cl–/HCO3– и Cl–-канал (и, возможно, как ко-транспортер анионов Na+) [33] и претендует, вероятно, на альтернативный путь ионной проводимости [32]. Кроме того, ассоциация МВСД, основанная на исследованиях генов-кандидатов СД2, была выявлена для вариантов TCF7L2, однонуклеотидных полиморфизмов (SNP — single nucleotide polymorphism) 3 генов CDKAL1, CDKN2A/B и IGF2BP2, которые, наряду с SLC26A9, определяют 8,3% фенотипического несоответствия МВСД и имеют совокупный популяционный риск 68% [32]. Генетическая модификация МВСД включает как общие, так и отличные от СД 2 типа пути. Больные МВСД, как правило, имеют нормальную чувствительность к инсулину и дефект секреции гормона β-клетки [34]. Хотя вторичная инсулинорезистентность формируется при остром (или обострении хронического) воспалении органов дыхания [35], применении глюкокортикоидов [20].

Инсулинотерапия является предпочтительным методом лечения МВСД с использованием стандартных базисно-болюсных схем введения, инсулиновых помп [1] и обеспечивает минимизацию долгосрочных микрососудистых осложнений [31]. Основаниями для выбора инсулинотерапии в качестве основной являются прогрессирующий абсолютный дефицит гормона β-клетки [34], анаболические эффекты экзогенного инсулина, противопоставленные усиленному катаболизму при хроническом инфекционном воспалении, часто используемой при МВ глюкокортикоидной терапии и, соответственно, данные исследований об успешности поддержания нормальной массы тела у больных [31].

Тем не менее альтернативные мнения исследователей [32] и данные систематического Кокрановского обзора [35] о методах оптимальной коррекции гликемии, ввиду отсутствия убедительных доказательств преимуществ инсулинов длительного или короткого действия перед пероральными гипогликемическими препаратами в контроле клинических исходов МВСД, также заслуживают внимания. Изучаются возможности применения ингибиторов дипептидилпептидазы-4 [7], агонистов рецепторов глюкагоноподобного пептида-1 [36] для улучшения секреции инсулина, сохранения массы β-клеток.

Интересно, что рецептор сульфонилмочевины-1, как и CFTR, является членом суперсемейства ABC-транспортеров и имеет значительную гомологию с ним. Выбор терапии в пользу производных сульфонилмочевины направлен на активацию и продолжительное открытие CFTR-канала, усиление движения Cl– через клеточную мембрану [19]. В единичном исследовании, посвященном этой теме, 45 пациентов с МВСД, принимавших инсулин или глибенкламид, не обнаружено различий в объеме жизненной емкости легких, но применение сульфонилмочевины коррелировало с улучшением качества жизни, снижением HbA1c, показатели гликемии были сопоставимы с таковыми в группе инсулина [37]. Изучение клинической эффективности терапии МВСД инкретинами, производными сульфонилмочевины у больных с сохраненной функцией β-клетки имеет главную цель и, возможно, перспективу — улучшение качества жизни, связанного с отсроченным стартом инсулинотерапии.

Новейшие препараты — таргетные корректоры и потенциаторы различных мутаций CFTR (малые молекулы, восстанавливающие процессы синтеза, транспорта к мембране или работу неполноценного белка CFTR), такие как Ivacaftor, Lumacaftor, нацеленные на наиболее распространенную мутацию F508del в гене CFTR в гомозиготном состоянии, в качестве протекторов дисфункции островкового аппарата ПЖ недостаточно изучены. Но данные об улучшении толерантности к глюкозе с восстановлением секреции инсулина на фоне их применения обнадеживают в перспективе терапии и профилактики МВСД [19].

## ОСТЕОПОРОЗ И МУКОВИСЦИДОЗ

Остеопения диагностируется у 38%, остеопороз — у 23% взрослых больных МВ, но очевидно, что уже в подростковом возрасте оптимальная пиковая костная масса недостижима. Переломы позвоночника развиваются у 14%, внепозвоночные — 19,7% детей и взрослых, по разным отчетам, частота переломов может достигать до 1/3 всех пациентов [34].

Дефицит и прогрессирующее снижение минеральной плотности костной ткани (МПК) являются ожидаемыми вторичными проявлениями МВ, развивающимися ввиду панкреатической экзокринной недостаточности с мальабсорбцией питательных веществ, кальция, жирорастворимых витаминов D и K (кофактора карбоксилазы, преобразующей неактивный некарбоксилированный остеокальцин в гамма-карбоксилированную активную форму). Усугубляется проблема дефицита набора костной массы с задержкой полового созревания и гипогонадизмом [1]. Наличие хронического инфекционного процесса, ведущее к системному воспалению, усиливает костную резорбцию. Известно, что риск остеопороза при МВ ассоциируется с полиморфизмом гена, кодирующего экспрессию воспалительного цитокина, фактора некроза опухоли-альфа (ФНО-α) [38], гена SLC6A14 (Na+ и Cl–-зависимый переносчик нейтральных и основных аминокислот) [39]. Влияние модифицирующих генов на процессы остеогенеза при МВ требует дальнейшего изучения [40].

В 2007 г. Shead et al. [41] указали на потенциальную первичную связь между мутацией CFTR и остеопорозом, обнаружив в срезах костной ткани экспрессию CFTR в остеобластах и остеоцитах, недавно включенных в кость. Более глубоко внедренные остеоциты, вероятно, не синтезируют белок хлорного канала. Остеокласты также снабжены CFTR, формирование его наблюдается в популяции гемопоэтических костномозговых клеток, но не выделено в пролиферирующих хондроцитах ростовых зон.

Степень ремоделирования кости определяется взаимодействиями между остеобластами и остеокластами. Лиганд-рецепторная система RANK/RANKL/OPG — ключевое звено гомеостаза кости, непосредственно регулирующее дифференцировку остеокластов и остеолиз. Основой этой системы является рецептор-активатор ядерного транскрипционного фактора NF-κB (receptor activator of nuclear factor kappa-B, RANK) — гомотримерный трансмембранный рецептор из семейства факторов некроза опухолей, экспрессируется остеокластами и его предшественниками. При этом NF-kB — универсальный фактор транскрипции, контролирующий экспрессию генов иммунного ответа, апоптоза и клеточного цикла. RANKL — лиганд, связывающийся с внеклеточным доменом RANK, трансмембранный протеин, синтезируемый и высвобождаемый остеобластами [42]. Слияние RANKL с RANK является главным активатором фактора транскрипции NF-kB, стимулятором дифференцировки и активности остеокластов, определяющим продолжительность их жизни и степень костной резорбции. Остеопротегерин (OPG) — гликопротеин, продуцируемый остеобластами, действует как растворимый рецептор, ингибирующий дифференцировку остеокластов и костную резорбцию, связываясь с RANKL и нейтрализуя его.

Одним из важнейших молекулярных сигнальных путей, ответственных за эмбриональное развитие, является Wnt-путь (объединенное название гомологичных генов насекомых — wingless и позвоночных — int). Wnt непосредственно регулирует биологический цикл стволовых клеток и тканевой гомеостаз, играет центральную роль в контроле над синтезом и дифференцировкой мезенхимальных стволовых клеток, а также репликацией преостеобластов, индукцией остеобластогенеза и подавлением апоптоза остеобластов и остеоцитов [43].

Лиганды Wnt могут стимулировать несколько сигнальных каскадов, в том числе канонический (Wnt/β-катенин), Wnt-кальциевый и др. пути. Канонический сигнальный путь отличается накоплением β-катенина в ядре клетки, который активирует факторы транскрипции (Т-клеточный фактор/лимфоидный фактор), модулирующие экспрессию генов-активаторов остеобластогенеза [43]. Синтез OPG также опосредуется сигнальным каскадом Wnt/β-катенин [44].

Неканонический Wnt-кальциевый путь не влияет на уровень β-катенина, регулирует полярность клетки, высвобождение кальция из эндоплазматического ретикулума для контроля за уровнем внутриклеточного кальция [43].

Простагландин (PG) E2 — главный продукт полиненасыщенных жирных кислот, производство которого катализируется циклооксигеназой-2 (COX-2) непосредственно в остеобластах. PGE2 и COX-2 представляют собой молекулы, необходимые для полной реализации сигналов Wnt. Концентрации PGE2 повышаются при снижении плотности кости. Воздействуя на собственные рецепторы 4 подтипа (EP4) на сенсорных нервах костной локализации, PGE2 повышает симпатическую активность и, далее, дифференцировку остеокластов и остеокластическую резорбцию в местах воспаления. По завершении образования лакун вновь зависимое от PGE2 снижение симпатической активности трансформируется в процессы пролиферации остеобластов, костную регенерацию [45]. Подобно паратгормону, хроническая стимуляция PGE2-рецепторов EP4 приводит к резорбции, в то время как периодическое воздействие является анаболическим. Оба пути запускают ремоделирование кости. Деятельность сенсорного нейрона, вероятно, отличает высокоточное пространственно-временное управление [45]. COX-2 и PGЕ2 рассматриваются как многофункциональные ключевые медиаторы костного обмена — от стадии воспалительной потери кости до дифференцировки ранних предшественников и окончательного созревания остеобластов [46].

У пациентов с F508del-мутацией, по данным Velard F. et al. [46], установлено значимое подавление синтеза в остеобластической формации OPG, COX-2, PGE2 (хотя в более ранних публикациях сообщалось об усилении продукции PGE2 при МВ [47]). То есть речь идет о дефекте пути передачи сигналов Wnt, разобщении этапности физиологического костного ремоделирования, возможно, компенсаторными адаптивными реакциями с последующим истощением этих механизмов (рис. 3).

**Figure:**
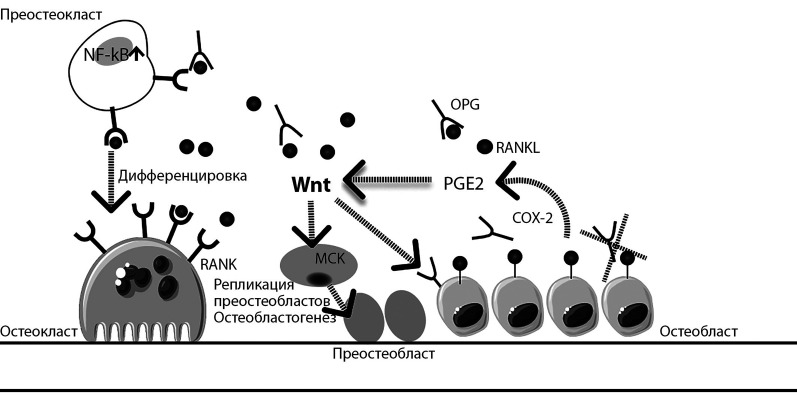
Рисунок 3. Многофакторный механизм нарушения костеобразования при наличии мутации CFTR (пунктирными стрелками и линиями показаны основные известные этапы в костном ремоделировании, патология которых развивается при дефекте транспорта ионов хлора).

Активация NF-κB в дифференцированных остеобластах оказывает антианаболический эффект. В эксперименте на животных с F508del-CFTR показано [48], что сигнальная и транскрипционная активность NF-kB значительно усилена в остеобластах, приводит к повышению фосфорилирования β-катенина со снижением его экспрессии. В целом мутация F508del CFTR нарушает дифференцировку и функцию остеобластов за счет ослабления передачи сигналов Wnt/β-катенина, подавления активности генов-мишеней остеобластогенеза.

Диагностика остеопороза — определение исходной МПК путем проведения двухэнергетической рентгеновской абсорбциометрии поясничного отдела позвоночника назначается детям старше 8 лет, взрослым пациентам (с 20 лет) рекомендуется исследование также проксимального отдела бедренной кости (анализируются область шейки бедра (Neck) и общий показатель бедра (TotalHip)) [1].

Данные периферической количественной компьютерной томографии высокого разрешения у больных МВ демонстрируют нарушение микроархитектоники кортикальной и губчатой кости [34]. Гистоморфометрия кости характеризуется уменьшением количества, снижением биосинтетического потенциала остеобластов и повышением численности и активности остеокластов с укрупнением резорбтивных поверхностей [49].

Терапия остеопороза базируется на оптимизации физической активности с индивидуальными программами расчета интенсивности упражнений; нутритивной поддержке препаратами кальция с оценкой статуса их абсорбции, витаминов D, К при поддержке заместительной терапии панкреатическими ферментами. При недостаточной эффективности вышеназванных мер оправдано фармакологическое вмешательство. К настоящему времени бисфосфонаты наиболее изучены в терапии остеопороза при МВ [50].

Систематический обзор девяти рандомизированных контролируемых исследований показал, что у больных МВ применение пероральных и внутривенных бисфосфонатов значительно повышает МПК. Но данные об уменьшении частоты переломов недостаточно убедительны [38]. Целью ближайших исследований является оценка эффективности лечения МВ-ассоциированного остеопороза деносумабом, предотвращающим активность RANKL и дифференцировку остеокластов. Улучшение показателей МПК регистрируется на фоне анаболической терапии терипаратидом, но возможности препарата в профилактике переломов требуют изучения [34].

## МУЖСКОЕ БЕСПЛОДИЕ И МУКОВИСЦИДОЗ

До 95% мужчин с МВ страдают обструктивной азооспермией по причине нарушения проходимости семявыносящих путей. Экспрессия дефектного белка хлорного канала приводит еще в эмбриогенезе к патологии развития мезонефротического протока и формирующихся из него семявыносящих протоков, семенных пузырьков и, как следствие, врожденной двусторонней или односторонней аплазии семявыносящих протоков (CBAVD — congenital bilateral aplasia of the vas deferens, CUAVD — congenital unilateral aplasia of the vas deferens). В общей структуре мужского бесплодия доля ассоциированных с мутациями гена CFTR причин составляет 1–2% и до 6% всех случаев обструктивной азооспермии [51]. Частота CUAVD может быть недооценена в связи с сохранностью одного семявыносящего протока [52].

При МВ могут отмечаться не полное отсутствие, а прогрессирующие с возрастом дегенеративные изменения vas deferens, ведущие к их гипо- или аплазии, тяжелой олигозооспермии или азооспермии без существенной патологии развития яичек. Следует различать изолированную CBAVD (CUAVD) у пациентов, не имеющих характерных для МВ симптомов [53].

Гипотеза о причине недоразвития протоков является аналогией аномальной секреции, обструкции и атрофии протоков ПЖ при дефекте хлорного транспорта. Предполагается, что CFTR играет роль в дифференцировке мезонефротических протоков, реализации собственно ветвления — фундаментального процесса создания канальцев, поддерживаемого ростовыми факторами и требующего участия консервативных сигнальных путей (в частности, Wnt/β-катенин [53, 54]), заключающегося в пролиферации, миграции, морфогенезе клеток, взаимодействиях посредством межклеточного матрикса [55].

В спектре мутаций CFTR у больных с классическим МВ и мужчин с синдромами CBAVD и CUAVD наблюдаются весомые различия. Так, для последних нехарактерно наличие двух тяжелых мутаций, например гомозиготности F508del, они несут по крайней мере одну копию CFTR с так называемой «легкой» мутацией [53]. Большинство пациентов (63–83%) с врожденной патологией vas deferens являются сложными гетерозиготами с составными мутантными аллелями, и около 43% пациентов с CUAVD несут по крайней мере один вариант CFTR. Среди этих аллелей наиболее распространены 5T, F508del и R117H [51].

Вариант полиморфизма политимидина (Tn) в интроне 9 IVS9-5T (вариант c.1210-7_1210-6delTT, ранее известный как IVS8-5T) (аллель 5T), последовательность из 5 тиминов в интроне 9 гена CFTR, что приводит к потере экзона 10 и образованию только 10% нормального белка. Этого достаточно для предотвращения патологий в других органах, обычно поражаемых МВ, но вызывает дисфункцию семявыносящего протока. TG(тимин-гуанин)-повторы ((TG)m) — непосредственно предшествующие Tn — также модулируют сплайсинг и могут быть причиной неполной пенетрантности аллеля 5T [53][51]. В отличие от аллеля Tn, который влияет на эффективность акцепторного сайта сплайсинга, аллели TGm изменяют положение ветви сплайсинга. Повторы TG от 9 до 13 увеличивают пенетрантность аллеля IVS9-5T и, следовательно, частоту, с которой экзон 10 удаляется во время сплайсинга [51]. Вариант 5Т в сочетании с (TG)12 или (TG)13 характеризуется более тяжелыми проявлениями изолированного CBAVD [53].

За последние 20 лет многочисленные исследования позволили охарактеризовать спектр мутаций CFTR у субъектов с CBAVD, указав их частоту в соответствии с этнической принадлежностью. В то время как одни и те же типы тяжелых мутаций обнаруживаются у пациентов с МВ, имеющих синдромы CBAVD и CUAVD, спектр мутаций CFTR при изолированной CBAVD радикально отличается. Существует множество не вызывающих МВ мутаций, большинство из которых может быть связано с другими фенотипами CFTR-зависимых состояний, такими как панкреатопатии, диссеминированные бронхоэктазы и синоназальные расстройства. Эти «легкие» мутации в основном включают интронные варианты, влияющие на сплайсинг, наиболее частым из которых является аллель 5T, и многочисленные миссенс-мутации, влияющие на функционирование хлоридного канала, наиболее частой у европейцев является мутация p.Arg117His (R117H) [53].

Большинство этих мутаций не выявляются стандартными панелями, разработанными для классической популяции МВ. Для молекулярной диагностики CBAVD рекомендуется применять исследование CFTR, включающее R117H и аллель 5T в качестве теста первой линии. В отсутствие результата проводится секвенирование всех экзонов и фланкирующих интронных областей CFTR, а также поиск крупных перестроек. Методы молекулярной диагностики, основанные на секвенировании нового поколения (NGS), все чаще используются для обнаружения не только точечных мутаций, но и крупных делеций или дупликаций [53].

Полиморфизмы в других генах могут увеличивать пенетрантность мутаций, связанных c CBAVD. К ним относят полиморфизмы генов Tr2GFB1 (трансформирующий фактор роста) и EDNRA (эндотелиновый рецептор типа A) [51].

Помимо CBAVD, увеличение частоты мутаций CFTR обнаруживается при необструктивной азооспермии, олиго-, астено- и тератоспермии [54]. Van der Ven et al. [56] провели скрининг на панель из 13 мутаций CFTR в образцах спермы инфертильных мужчин. 14 (17,5%) из 80 пациентов с бесплодием из-за снижения качества спермы, 2 из 21 (9,5%) — с азооспермией несли одну мутацию CFTR, в то время как ген не был дефектен у 26 мужчин с нормозооспермией. Потребность познания роли CFTR в мужской репродуктивной физиологии, участие анионного транспорта в сперматогенезе дали старт новым исследованиям.

Сперматогенез осуществляется в семенных канальцах и представляет собой сложный процесс деления плюрипотентных стволовых клеток с последующей их морфологической и функциональной дифференцировкой до зрелых сперматозоидов. Герминативные развивающиеся клетки находятся в тесном контакте с так называемыми «поддерживающими» соматическими клетками (клетки Сертоли, сустентоциты), обеспечивающими метаболическое сопровождение гаметогенеза. Исследование экспрессии белка хлорного канала CFTR и регулируемого им ENaC с помощью конфокальной микроскопической иммунофлуоресценции в срезах семенных канальцев показало, что во всех фазах развития половых клеток (сперматогонии, сперматоциты и сперматиды) ENaC локализовался в цитоплазматических пулах, а также вдоль хвостов сперматид. В сперматозоидах, выделенных из придатка яичка, ENaC был выделен на акросоме и в центральной области жгутика, и его расположение указывает на роль в преодолении сперматозоидом блестящей оболочки ооцита. В отличие от ENaC, иммунофлуоресценция самого канала CFTR наиболее отчетливо представлена только в ядрах клеток Сертоли. Вероятно, CFTR выполняет роль не только ионного транспортера, но и независимого регулятора активности генов сустентоцитов [57]. Хлоридный канал участвует в поддержании нормального цитоскелета клеток Сертоли [58].

Фолликулостимулирующий гормон (ФСГ) после связывания c собственными рецепторами на клетках Сертоли стимулирует мембраносвязанную аденилатциклазу (mAC), которая активирует цАМФ-зависимую протеинкиназу А (cAMP/PKA) и далее фактор транскрипции CREB (cAMP response element-binding protein). В 2000 г. Chen et al. клонировали новый тип аденилатциклазы, названный растворимой аденилатциклазой (sAC). Он заметно отличается от mAC тем, что чувствителен к HCO3– и кальцию и представляет собой альтернативный способ активации cAMP/PKA. Этой же группой ученых было продемонстрировано, что CFTR участвует во входе HCO3– в клетки Сертоли для запуска cAMP-зависимой транскрипции и, в конечном итоге, обеспечения сперматогенеза. Дефекты CFTR могут приводить к недостаточности ФСГ-опосредованной внутриклеточной передачи сигнала и, как следствие, экспрессии генов сперматогенеза [54]. Так, отсутствие синтеза CFTR у пациентов с необструктивной азооспермией отличается дефицитом фосфорилирования CREB, его суммарной экспрессии в клетках Сертоли [59]. Вопрос о факторах прогноза по угнетению гаметогенеза с возрастом у мужчин с мутациями CFTR требует изучения (рис. 4).

**Figure:**
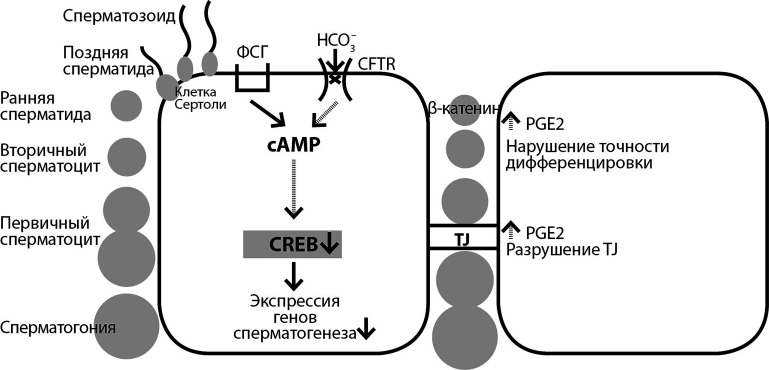
Рисунок 4. Механизмы развития патологии сперматогенеза при мутации CFTR(пунктирными стрелками обозначены внутри- и межклеточные реакции и процессы, изменяющиеся при дефекте транспорта ионов хлора)

Плотные запирающие контакты (tight junctions-TJ) между соседними клетками Сертоли составляют гематотестикулярный барьер (ГТБ) — регуляторы обмена веществ между кровеносными сосудами и семенными канальцами, формирующие микроокружение для дифференцирующихся половых клеток. Состоятельность TJ, по крайней мере частично, контролируется цитокинами, высвобождаемыми Сертоли и/или зародышевыми клетками. Доказано, что CFTR значимо подавляет путь NF-κB/COX-2 и продукцию PGE2 в дыхательных путях. Однако ингибирование CFTR в клетках Сертоли приводит к чрезмерной активации NF-κB и производству PGE2 с разрушением TJ белков, к примеру, окклюдина, что снижает трансэпителиальную резистентность, нарушая ГТБ [54].

Функциональное значение локализованного в ядре сперматоцитов и круглых сперматид β-катенина считается показателем статуса активации пути Wnt в развивающихся половых клетках. Точность контроля передачи сигналов Wnt важна для смены стадий нормального сперматогенеза [60]. Колебания продукции и концентраций компонентов Wnt, вероятно, определяют патологические исходы развития и дифференцировки зародышевых клеток сперматогенеза при МВ.

Сведения о глубоком угнетении процессов дифференцировки сперматозоидов при несостоятельности белка хлорного канала позволяют задуматься о важности дальнейших исследований зависимости поражения клеток Сертоли, половых клеток и непременного либо, напротив, избирательного развития необструктивной азооспермии от тяжести или типа мутации CFTR, возраста пациентов, наличия дефектов модифицирующих генов.

При использовании вспомогательных репродуктивных технологий, в частности, аспирации сперматозоидов из яичек или их придатков, внутрицитоплазматической инъекции сперматозоидов и экстракорпорального оплодотворения, мужчины с CBAVD и сохраненным сперматогенезом могут иметь потомство. Обязательным для таких пациентов является генетическое консультирование с целью привлечения внимания к вероятности рождения потомства, несущего дефект CFTR.

## ЗАКЛЮЧЕНИЕ

МВ — заболевание, характеризующееся поражением экзокринных желез, классическими проявлениями которого являются обструктивный бронхит, кистозная трансформация ПЖ, аплазия семявыносящих протоков у мужчин. СД, остеопороз принято относить к осложнениям данного заболевания. Исследования последних лет позволили изучить регуляцию белком хлорного канала основных биологических механизмов ряда эндокринных клеточных комплексов, в частности, островкового аппарата ПЖ, костной ткани, сперматогенного эпителия. Доказано, что наличие мутаций CFTR ассоциируется с прогрессирующими функциональными и структурными повреждениями β- и α-клеток ПЖ, дисфункцией и дефектом секреции инсулина и глюкагона, а также развитием остеопороза ввиду первичного подавления остеобластогенеза. Патология белка CFTR характеризуется дезорганизацией жизненного цикла созревания сперматозоидов на любом из его этапов по причине несостоятельности клеток Сертоли и ГТБ и формированием в конечном итоге, наряду с обструктивной, необструктивной азооспермии. Таким образом, МВ как болезнь полиэндокринных нарушений заслуженно привлекает внимание эндокринологов. Выявление МВ в группах риска пациентов различного возраста и пола на доклинической стадии имеет целью предупреждение поздних осложнений заболевания, увеличение продолжительности и качества жизни больных.
